# Editorial: Women in gastrointestinal and hepatic pharmacology: 2024

**DOI:** 10.3389/fphar.2026.1838196

**Published:** 2026-04-01

**Authors:** Ariane Leite Rozza

**Affiliations:** Department of Structural and Functional Biology, Institute of Biosciences, São Paulo State University (Unesp), Botucatu, Brazil

**Keywords:** acute intestinal injury, gender equity, obesity, steatosis, ulcerative colitis, women in science

## Introduction

Gender inequality remains a persistent challenge across science, technology, engineering, and mathematics (STEM), including within the scientific research enterprise itself. Recent global analyses indicate that, although women represent a substantial proportion of university graduates and nearly half of doctoral degree holders, they remain underrepresented in the research workforce and are less likely to occupy senior academic positions, reflecting a well-established “leaky pipeline” (European Research Executive Agency, 2026). Structural factors such as implicit bias, unequal access to funding and leadership opportunities, and the cumulative impact of caregiving responsibilities continue to shape academic trajectories. These disparities are driven by systemic inequities embedded within academic culture and evaluation systems (Cheryan et al., 2025).

Within the life sciences and health-related fields, this pattern persists, with women less frequently represented in positions of authority, senior authorship, and decision-making roles. Studies examining scientific authorship and productivity demonstrate that women remain underrepresented as first and last authors, reinforcing disparities in visibility and recognition (Hennessey et al., 2026). Concurrently, a growing body of evidence highlights that diverse scientific teams foster greater innovation, creativity, and problem-solving capacity, underscoring that gender equity is not only a matter of fairness but also a key driver of scientific excellence (García-Silva et al., 2025). Addressing these imbalances requires institutional efforts and structural reforms aimed at promoting equity, diversity, and inclusion across all levels of the scientific workforce.

In this context, initiatives that actively promote the visibility and leadership of women in science are essential. The Research Topic “*Women in Gastrointestinal and Hepatic Pharmacology: 2024*” in Frontiers in Pharmacology represents a meaningful contribution to this effort by showcasing the scientific excellence and diversity of women researchers in this field. In this Research Topic, three original research articles and one study protocol were published.

The study conducted by Alioui et al. demonstrated that *Cantharellus cibarius* Fr., popularly known as the golden chanterelle mushroom, may represent a novel therapeutic approach for treating ulcerative colitis. Using a murine model of DSS-induced colitis, in which mice received drinking water containing 3% DSS for 7 days, the authors showed that intestinal integrity was preserved, the expression of pro-inflammatory cytokines decreased, anti-inflammatory cytokines were upregulated, and gut dysbiosis was modulated following supplementation with *C. cibarius* Fr. crude extract. These findings are particularly relevant, as the prevalence of ulcerative colitis was estimated at 5 million cases worldwide in 2023, with incidence continuing to increase annually (Le Berre et al., 2023).

Emilio-Silva et al., in a study supervised by Hiruma-Lima, investigated the beneficial effects of citral in high-fat diet-induced obesity in mice. *In vivo*, oral administration of the monoterpene citral for 17 weeks resulted in reduced body fat, glycemia, and cholesterol levels, in addition to decreasing serum levels of endotoxins and cytokines. The anti-obesogenic, anti-inflammatory, and anti-hyperlipidemic properties of citral position this monoterpene as a promising candidate for mitigating the harmful effects of obesity. According to the World Health Organization, more than 1 billion people worldwide are currently living with obesity, including children, adolescents, and adults (NCD Risk Factor Collaboration, 2024). Therefore, identifying molecules capable of attenuating its deleterious health effects, such as dyslipidemia and chronic inflammation, remains of critical importance.

Metabolic dysfunction-associated steatotic liver disease is one of the leading causes of liver-related morbidity and mortality. In this regard, the study conducted by Li and Jiao sought to bridge basic and clinical research by validating the efficacy of the G protein-coupled estrogen receptor 1-specific agonist (G1) in improving hepatic steatosis associated with metabolic dysfunction in humans. Using both *in vitro* approaches (a free fatty acid-induced hepatocyte steatosis model) and *in vivo* analyses (gene expression in human liver samples), the authors demonstrated that G protein-coupled estrogen receptor 1 expression is reduced in affected individuals and is associated with increased severity of steatosis. Conversely, administration of the agonist resulted in improvements in hepatic steatosis through multiple mechanisms mediated by G protein-coupled estrogen receptor 1, including the regulation of lipid metabolism and the reduction of oxidative stress and apoptosis in the liver. The selective activation of this receptor therefore emerges as a potentially safe therapeutic alternative for liver protection.

In this Research Topic, we also welcomed a study protocol. Based on the observation that patients undergoing pelvic radiotherapy frequently develop acute intestinal injury, Li et al. proposed a study protocol consisting of a multicenter randomized clinical trial to investigate the efficacy of glucocorticoid therapy in the treatment of radiation-induced acute intestinal injury. The authors propose allocating 60 patients into two groups to compare the efficacy of conventional therapy alone versus its combination with dexamethasone enemas. The primary objective is to assess mucosal recovery and improvement of clinical symptoms, such as abdominal pain and diarrhea. In addition to efficacy, the study will rigorously monitor safety and potential adverse effects associated with glucocorticoid use. This protocol may provide a scientific basis for standardizing the therapeutic management of radiation-induced acute intestinal injury following pelvic radiotherapy.

Finally, as an editor of this Research Topic, I am grateful to all women scientists who contribute to scientific, technological, and societal development, inspiring one another and future generations of scientists, and advancing gender equity in science.

## Conclusion

This Research Topic highlights both scientific advances and the essential contributions of women in gastrointestinal and hepatic pharmacology. Initiatives like this are key to increasing visibility, promoting equity, and fostering a more inclusive and innovative scientific community.
